# Developing program theory for purveyor programs

**DOI:** 10.1186/1748-5908-8-23

**Published:** 2013-02-19

**Authors:** Christa Oosthuizen, Johann Louw

**Affiliations:** 1 Department of Psychology, University of Cape Town, PD Hahn Psychology Building, (Chemistry Mall), University Avenue, Cape Town, South Africa

**Keywords:** Program theory, Logic models, Purveyor, Dissemination, Implementation fidelity

## Abstract

**Background:**

Frequently, social interventions produce less for the intended beneficiaries than was initially planned. One possible reason is that ideas embodied in interventions are not self-executing and require careful and systematic translation to put into practice. The capacity of implementers to deliver interventions is thus paramount. Purveyor organizations provide external support to implementers to develop that capacity and to encourage high-fidelity implementation behavior. Literature on the theory underlying this type of program is not plentiful. Research shows that detailed, explicit, and agreed-upon program theory contributes to and encourages high-fidelity implementation behavior. The process of developing and depicting program theory is flexible and leaves the researcher with what might be seen as an overwhelming number of options.

**Methods:**

This study was designed to develop and depict the program theory underlying the support services delivered by a South African purveyor. The purveyor supports seventeen local organizations in delivering a peer education program to young people as an HIV/AIDS prevention intervention. Purposive sampling was employed to identify and select study participants. An iterative process that involved site visits, a desktop review of program documentation, one-on-one unstructured interviews, and a subsequent verification process, was used to develop a comprehensive program logic model.

**Results:**

The study resulted in a formalized logic model of how the specific purveyor is supposed to function; that model was accepted by all study participants.

**Conclusion:**

The study serves as an example of how program theory of a ‘real life’ program can be developed and depicted. It highlights the strengths and weakness of this evaluation approach, and provides direction and recommendations for future research on programs that employ the purveyor method to disseminate interventions.

## Background

In many resource-poor countries, governments and non-governmental organizations struggle with what is generally referred to as ‘lack of capacity.’ This is typically seen as a basic human resource issue: there are inadequate skills and expertise in an organization to perform the required tasks, and in addition, the organization has inadequate governance and management structures to support its personnel [1,2]. A variety of organizations, referred to as purveyors and intermediary organizations, have been developed to address these problems in program implementation.

Purveyors are individuals or organizations that operate as outside experts representing a particular program; they support organizations, systems, and practitioners in striving to adopt and implement that program with fidelity [3]. They are typically involved in specific programs or practices, while intermediary organizations tend to have a broader role in the support of multiple programs; that role generally entails building capacity within a system or agency [4]. Provider organizations adopt interventions and employ a group of individuals, also known as implementers, who deliver the intervention to the intended beneficiaries [5]. In this article the term ‘purveyor programs*’* refers to the support services delivered by purveyors.

Purveyors and intermediary organizations are receiving increasing attention in implementation science. In August 2011, the first Global Implementation Conference [6] was held in Washington, DC, and included a plenary session on purveyors. That conference is a good source of information on practices and the science related to implementation, and was attended by representatives from a number of organizations active in the field^a^. A group known as the Practice Group for Purveyors and Intermediary Organizations [7] grew out of the Washington conference.

Authors such as Elliot and Mihalic [8], Fixsen, Blasé, Naoom, and Wallace [5], and Fixsen, Naoom, Blasé, Friedman, and Wallace [1] have made significant contributions to the field. Moreover, the National Implementation Research Network [9] has developed a number of core implementation components, which have been applied to purveyors, especially by Fixsen *et al.* Research has tentatively identified some core factors deemed necessary to ensure high fidelity implementation of interventions [10]. These activities are known as core implementation drivers and include both technical (site selection, training sessions, consultation and coaching, staff and program evaluation) and management capacity (facilitative administrative support and systems interventions) [1,8]. The work of purveyors is made more complicated because their support has to be assimilated by provider organizations. This is an important area for further research, where we still lack information about effective procedures [11,12]. Fixsen *et al.* singled out inconsistent implementation strategies and procedures employed by purveyors as a characteristic of the field at present [5]. One significant finding is that ‘augmented products’ that could include a combination of customisation, training, coaching, manuals, and a help desk, are adopted more easily by provider organizations and implementers [12].

These caveats notwithstanding, the purveyor method, in its various shapes and forms, has been applied to a wide variety of human service fields to disseminate interventions: these fields include education [13], juvenile justice [14], substance abuse [15], family support [16], medicine [17], nursing [18], mental health [11], and social work [19].

### The program

Three years ago, we became involved in the work of a South African non-governmental organization (NGO) that acts as a purveyor to seventeen local organizations that deliver a peer education intervention to young people as an HIV prevention intervention. It was not easy to classify this NGO as a purveyor or intermediary organization, in the light of the definitions given above. It contains elements of both types of organization, but eventually a decision was taken to classify it as a purveyor, because it provides external expertise, and works with one particular type of program (peer education).

It is widely known that South Africa faces a serious HIV/AIDS problem: in 2009, HIV prevalence among adults aged 15 to 49 was calculated at 17.8% of this population, with the number of adults and children aged 0 to 49 living with HIV estimated at 5,600,000 [20]. Peer education is a promising approach that is believed to have a positive effect on sexual behavior among youth [21-23]. It is an approach that has been used increasingly over recent years, especially by interventions in the field of youth HIV prevention and sexual health [24,25]. It is currently one of the most important ingredients in preventive, supportive, and educational interventions [26], despite some doubt about its effectiveness [27].

The seventeen provider organizations work in 102 schools in South Africa and Botswana, and have trained more than 5,000 peer educators. At the time of the study, the purveyor (from here on the agency) had been in existence for three years, with a growing demand for its services. Currently, it has an international office in the Western Cape and four provincial offices. The international office supports the provincial offices in their efforts to provide ongoing technical assistance and support to the provider organizations and implementers in the respective provinces. The director is based at the international office, and each province has a provincial manager in charge of its activities. The agency’s purveyor program is divided into the following six functional areas, each with a manager in charge to ensure that services are delivered consistently to all provider organizations and implementers:

1. Advocacy and visibility—develops and distributes resources, knowledge, and skill to promote the peer education intervention.

2. Quality assurance—provides knowledge and skill to monitor delivery of the peer education intervention.

3. Research and development—develops and distributes all resources required to implement the peer education intervention.

4. Resource mobilization—develops and distributes resources, knowledge, and skill to help provider organizations to obtain resources independently.

5. Stakeholder management—ensures that an increasing number of organizations deliver the peer education intervention. It also ensures that current provider organizations and implementers have opportunities to interact to ensure that they do not feel isolated.

6. Training and support— provides implementers with the knowledge and skill needed to deliver the peer education intervention with fidelity.

Although the original evaluation approach was formulated in terms of an implementation evaluation of the purveyor program, our initial contact with the agency revealed a problem that needed addressing before we could embark on an implementation study. In program evaluation terms, the agency had no explicit and agreed-upon program theory. To put it differently, it became apparent to us that it was not clear to the stakeholders how the purveyor program should work.

### Program theory

Program theory-based evaluation is a well-known evaluation approach [28], and does not need extensive coverage here. Rossi, Lipsey, and Freeman [29] called it ‘the set of assumptions about the manner in which the program relates to the social benefits it is expected to produce and the strategy and tactics the program have adopted to achieve its goals and objectives.’ According to two prominent authors in the field, Donaldson and Lipsey [30], it has become more valued and common to depict program theory in evaluations.

Funnel and Rogers [31] argue that program theory could be especially useful for interventions that employ purveyor programs because so many organizations are involved. They used this theory, for example, to develop service agreements and specifications for what the programs should entail. In his paper dealing with taking programs to scale, Baker [32] called program theory the first ‘pre-exploration’ phase in enlarging a program, in which a clear logic model must be developed. In addition, the exercise of making implicit assumptions explicit often exposes faulty thinking on the part of the original program developers, which can subsequently be corrected to improve the conceptual base of programs [33]. Also, a common understanding of how the program is meant to work encourages program staff members to work together and to focus on those activities that are most important for program success [34].

Funnel and Rogers [31] identified four clusters of program theory use. These are: to aid in planning or preplanning an intervention; for managing and engaging stakeholders; for monitoring and evaluation; and for evidence-based practice by documenting innovative practices and supporting adaptation of program elements. As the agency had already been providing services for three years when the present study started, the first cluster identified by Funnel and Rogers did not apply. The benefits we envisaged were in line with the other three clusters of theory use. It would clarify the specific mechanisms involved in the purveyor program that result in the following: a shared understanding of the purveyor program among stakeholders; a more effective and efficient monitoring system; and a solid basis for any future evaluation of the agency’s work. It was argued that this process would not only encourage and increase the commitment, focus, and effectiveness of the agency’s staff members, but would also ensure that essential components of the purveyor program are clearly defined and remain relevant when the agency expands its service delivery.

A decision was therefore taken, in close consultation with program management, to articulate the ‘theory’ underlying the purveyor program; in other words, the understanding of the way it is supposed to work, both in terms of process and outcome. This was thought to be an important exercise, because it was expected that the agency’s purveyor program would be extended to other provider organizations in the local context.

Thus, the primary focus of the study was to unfold the theory of change underlying the agency’s purveyor program. On a practical level, it was argued that this would result in a better understanding of the purveyor program among the agency staff members, and would consequently encourage the strength and fidelity of implementation of the purveyor program to current and future provider organizations and implementers. Less modestly, we also aimed to furnish information that could contribute towards a better understanding of purveyor programs in general. This study also serves as an example of how a detailed, explicit, and agreed-upon representation of program theory could be developed and depicted. In other words, it is a relatively small contribution to the burgeoning field of implementation science and practice.

## Methods

The information used to construct program theory can be obtained from multiple sources, including the following: a review of program documentation; interviews with those closest to the program; observation of the program; prior theory and research in the specific program domain; or exploratory research that tests the critical assumptions of the program [28].

The first decision to be taken was who should be involved in developing the program theory of the purveyor program [31]. The method of extracting program theory can vary from cases where the evaluator largely takes the responsibility of developing the program theory to cases where it is developed solely by those closest to the program [33]. Various evaluation practitioners describe the best approach for extracting and developing program theory as lying somewhere between these two extremes [28,35,36]. Program staff and stakeholders hold essential context-specific information on how programs ought to prevent or ameliorate social problems. The evaluator’s knowledge of social science theory and access to prior research, if this exists, can be applied to develop further and assess the feasibility of the program theory as it is being developed [28]. In addition, involving evaluation stakeholders during the design and implementation phases of the evaluation increases their buy-in to the evaluation and also facilitates understanding of the evaluation processes; both of these factors have been shown to contribute significantly to the relevance and use of the evaluation processes and findings [37].

There are three main approaches to developing program theory: deductive; inductive; and user-focused [35]. The deductive approach relies exclusively on empirical research and employs dominant theories of various disciplines to inform theory development. The inductive approach requires the evaluator to generate the program theory by observing the program in action through fieldwork and review of program documentation, while the user-focused approach requires the evaluator to obtain information from program staff that is then used to construct the program theory.

Due to the scarcity of literature and general lack of knowledge about the program theory underlying purveyor programs, the authors opted to combine the inductive and user-focused approaches. The first author took the lead role, with the second author acting as evaluation consultant, which implied that it fitted an approach where evaluators facilitate a collaborative process of developing the program theory [38]. Given time and resource constraints, a decision was taken to involve only those closest to the purveyor program; in other words, individuals who were sufficiently knowledgeable to act as reliable sources of information. Only staff members of the agency participated in this exercise. These were the director, eight senior managers, and two provincial and six functional area managers.

The development of logic models usually involves the evaluator constructing a preliminary draft based on available program documentation, which is then presented to program staff members for validation. This results in an iterative process of moving back and forth between the development of the logic model and receiving feedback from staff members, which will continue until all staff members agree that the model is an accurate and detailed description of the program as it was originally intended [29,39].

Numerous site visits to the international office served to develop familiarity with the agency, to establish contact with program staff, to observe the program as it is being delivered, and to identify useful documentation that should be included in the review. Relevant documents were identified and studied, including operational plans, the implementation manual, peer educator portfolios, annual reports, quality assurance mid-year assessments and the like. These documents were analyzed and data sorted into coding categories according to standard logic model components. As a result, we ended up with a preliminary working understanding that covered the following categories: inputs; activities; outputs; pivotal proximal and intermediate outcomes; and distal outcomes.

This preliminary logic model was then submitted via a series of relatively unstructured interviews to the individuals selected to participate in the exercise. Two main topics structured these interviews: first, what the goals and objectives were for the specific program areas that these individuals managed; and second, how these aims contributed to the overall effectiveness of the program. Thus, an iterative process was started between our observations, the program documentation, and data resulting from the interviews and the subsequent verification process; this ultimately culminated in a final program logic model. Each iteration resulted in a more refined and accurate model of the program. It took four months to move from initial data gathering to reach the point at which all the participants were satisfied that the model accurately and comprehensively represented how the agency aims to affect change within the provider organizations and implementers.

In summary: we developed the program theory of the purveyor program from implicit theories of those closest to the program, observations of the program, and program documentation.

The study was approved by an Ethics Review Committee of the Faculty of Humanities at the University of Cape Town. The director of the agency supported the study and communicated its purpose and usefulness to the various evaluation stakeholders and urged them to cooperate. No one refused to participate in the study.

## Results

**Figure 1:**
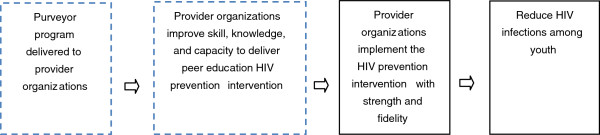
**General program theory underlying purveyor programs. **A diagrammatic representation of the relationship between purveyor programs, the proposed intervention, and the expected impact of this intervention. Although the direct involvement of the purveyor is limited to the first two blocks with dotted outlines, it is assumed that the supportive services will result in higher levels of implementation fidelity that in turn will lead to the expected impact of the intervention.

Through an iterative process of logic model development, refinement via interviews, and further presentations of the revised logic model, we arrived at the major result of this study: a formalized logic model of the purveyor program’s theory which was accepted by all concerned as an accurate reflection of the work done by the agency. The challenge however is to present such a complex program visually, in a way that is useful to the stakeholders, comprehensible and engaging to those less familiar with it, communicates effectively, and simplifies without trivializing [31]. We decided to build a cascading visual model of the program theory, starting with the general logic of purveyor programs. As indicated above, the central idea behind purveyor programs is that they provide external systems of support to increase capacity in provider organizations, which in turn will support high fidelity behavior in implementers. Figure 1 captures this overall logic.

Note, in particular, that the present exercise refers only to the two boxes on the left of Figure 1, in dotted outline. In other words, for the purveyor, an increase in the indicated capacities is an outcome of note, but further down the outcome chain, the provider organizations and implementers must bring about the ultimate benefits of this effort—*i.e.*, the reduction of HIV infections among young people. Thus, the purveyor may be improving the capacity of implementing organizations to deliver the peer education intervention as intended, but it remains an open question whether the latter actually do implement peer education with fidelity and strength. At the time of our involvement, the agency had focused its monitoring and evaluation efforts on how provider organizations and implementers deliver the peer education intervention, with little attention given to monitoring the agency’s supportive activities. Although this is understandable, it fails to recognize the extent to which subsequent steps (the two boxes on the right: intervention implementation, outcome, and impact) depend on getting fidelity and strength in the agency’s own activities.

**Figure 2:**
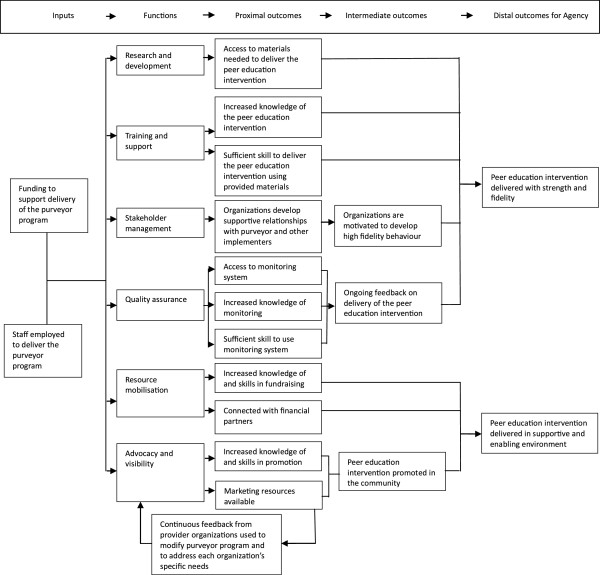
**Program theory underlying a specific purveyor program. **A diagrammatic representation to explicate the logical linkages between the specific elements of the purveyor program and the expected proximal, intermediate, and distal outcomes.

The next step was to elaborate on these two boxes that represent the purveyor program. One way to deal with this complexity is to use sub-pages, as is the case in visual outcomes, model- building software such as DoView [40]. In other words, below this level or page depicting the general program theory for purveyor programs, there are further pages, depicting the logic behind a particular program element. We show this in Figure 2, providing a more detailed description of the assumed progress between each functional area and the distal outcomes of the purveyor program.

Of course, this model is still fairly general, because each functional area has a distinctive service delivery plan that includes various activities deemed necessary to result in the function’s expected outcomes. In other words, another sub-page that depicts these aspects is required. We went one step further for the agency, and developed the program theory underlying the activities of each functional area. These six separate logic models are not presented here, as we are more interested in the general principles rather than in the specific details of the purveyor program.

The theory flows from left to right, starting with the six program functions. The activities of each function are described below. These activities are presumed to lead to short-term, early changes, which in turn set in motion changes in the medium term, which are expected to result in a distal outcome as indicated.

The advocacy and visibility function includes an annual activity to promote the agency’s work, but invests most of its time and resources in developing and distributing marketing resources. Provider organizations receive the marketing material from the agency, and this is intended to assist them in promoting the peer education intervention in the relevant communities.

The quality assurance function offers provider organizations a standard monitoring and evaluation system to monitor delivery of the peer education services. The system is based on a Logical Framework Analysis that provides standards and guidelines for implementation practices. Implementers have the skills and knowledge needed to use the system, and they gather information to send to the agency’s quality assurance coordinator at the international office. There, the incoming data is analyzed to identify patterns of delivery to provide systematic feedback on the provider organizations’ performance in comparison to quarterly targets.

In terms of research and development, training resources such as the training sessions and workshops are examined and modified if necessary. Research also is conducted with stakeholders to update, for example, the implementation guide. Thus, implementers have easy access to resources that are continuously examined for its relevance and usefulness in those communities where the provider organizations deliver the peer education intervention.

The resource mobilization function offers funding to provider organizations and connects them with reliable and sustainable financial partners. In addition, it spends a significant amount of its time and resources on activities to equip provider organizations with the skills and tools needed to obtain their own resources, independently from the agency. These activities include the development and distribution of an information pack, the delivery of a workshop, and the development and distribution of a list of potential donors.

The stakeholder management function has the role its title suggests: it identifies and recruits new provider organizations, and maintains the collaboration of current provider organizations with the agency. Particular care is taken to increase the supportiveness of the environment in which the peer education intervention is delivered, via efforts such as peer education forums and peer education workshops.

The training and support services offered to provider organizations include training sessions, workshops, mentoring, and coaching for the implementers. The purpose is typical of such interventions: to equip implementers with the skills and support deemed necessary to ensure effective delivery of the peer education intervention. The training function is responsible for delivering eleven training sessions and workshops, and it conducts bi-annual on-site visits and offers continuous telephone and email support to all the implementers.

## Discussion

This study was launched by the realization that the agency lacked an explicit model or theory of how the purveyor program was supposed to work, and what it was supposed to achieve. This was even though it had been running for three years, and with a growing demand to extend its reach to include more provider organizations. In practical terms, our first objective was to develop such a theory for the purveyor program and its staff. This was regarded as an important precursor to further evaluation studies and in particular, a possible process and impact evaluation.

As indicated earlier, we also had a somewhat more ambitious objective in mind: to use this particular instance of program theory development as a tentative indication of how one could depict purveyor programs. These programs are complex, because they involve multiple implementation sites, and many provider organizations: in this study, 17 provider organizations, with the possibility of expanding this number. Purveyor programs of this kind present an interesting challenge to evaluation, and to program theory-building in particular. It was exactly this realization that diverted our attention from an implementation assessment to a theory-building exercise in the present study.

In terms of the actual supportive activities carried out by the agency, it is a relatively simple program: all the functions and their associated activities are well-defined and understood, and delivered in a reasonably consistent and even manualized manner by the agency. Nevertheless, these are all delivered at very different sites, and to provider organizations with very different governances. This presents a challenge in terms of understanding the purveyor program, what the expectancies of various shareholders are, what local variations can be tolerated, and so on. For present purposes, we thought an understanding of the causal path underlying the intervention was particularly important.

Thus, the present study focused on those activities and outcomes involving the agency, as depicted in Figure 1, and not the provider organizations. Because of multiple functions contained in the purveyor program, one has to expect multiple simultaneous causal strands. In an attempt to address this, we followed a stepped approach to theory development: first the overall program logic was developed as shown in Figure 1, then one which disaggregated the purveyor program in terms of different functions (Figure 2), and finally, the logic of each individual function (not dealt with in this study). It is our impression that the logic models that emerged as a result of this exercise were useful to evaluators, agency stakeholders, and that these clarified their thinking about the purveyor program considerably.

If, however, the agency wishes to understand and/or investigate what happens in the provider organizations themselves, after their involvement with the agency (the two boxes on the right in Figure 1), the situation very quickly becomes much more complicated. Suppose the agency, for example, wishes to address the question: ‘Does increasing capacity in 17 provider organizations lead to the peer education interventions being implemented at strength and with fidelity?’ In this instance, every one of these organizations will have to be studied quite carefully. This is the attribution challenge faced by programs that employ the purveyor method to disseminate interventions, and it is our contention that drawing out an explicit logic model helps the agency to come to grips with it. A first step could be the development of a logic model for each of the 17 provider organizations, describing the theory of change, and the activities they carry out to achieve their goals. Figure 1 makes it clear that overall the agency and its provider organizations share the same program goals, even though it is likely that they follow different paths to get there. Indeed, such an exercise will yield useful comparative information about the provider organizations, and the breadth and depth of their activities.

Starting with documentation, we repeatedly submitted the emerging program theory to the agency’s staff members for consideration. This recursive way of working is in line with how program theory typically is drawn out, and it resulted in four major benefits. All of these benefits are likely to improve the usefulness and utility of subsequent evaluations of the purveyor program.

First, it allowed for a clear articulation of the activities that make up the purveyor program. In many instances it was not clear what the activities of the agency include, and where the boundaries of the different components were. The importance of good program descriptions is widely acknowledged, especially in the light of the present program’s ambition to be scaled up. The present theory-driven approach filled that gap in this specific purveyor program, and elicited the causal assumptions underlying these activities.

Second, it allowed us ample time to spend with the agency’s staff members, and to become thoroughly familiar with the purveyor program. Rossi, Lipsey and Freeman [29] have indicated that this process builds a knowledge base about the program, which enabled us to develop a detailed description of what supposedly occurs between the intended activities and the expected benefits of the purveyor program. Indeed, literature suggests that stakeholder input increases, substantially, the evaluation’s relevance and usefulness [41]. Interactions with the staff members also provided an opportunity to implement various strategies to manage and overcome evaluation anxiety. These strategies included explaining the purpose of the evaluation, allowing stakeholders to discuss and affect the evaluation, and distinguishing between program and personnel evaluation. If not addressed adequately, evaluation anxiety could have consequences that range from reduced utilization of evaluation findings to problems with compliance and cooperation [42].

Third, it helped us to become familiar with the context in which the agency operates. Literature suggests that this not only will facilitate the development of the data collection tools and the interpretation of the evaluation findings, but it will also greatly increase the evaluation’s relevance and usefulness.

Finally, the theory development process can, quite easily, be employed to ensure that evaluations are conducted only on programs that meet evaluability assessment criteria [43]. For example, it enabled the present study to ascertain whether the agency had well-defined and plausible goals and objectives, and whether relevant performance data could be obtained at reasonable cost.

Although a program theory-based approach has substantial benefits to evaluation, it challenges the researcher in at least two ways. For a start, one is confronted with a relative lack of clarity on just what is meant by ‘program theory’ and there is a scarcity of examples of applications using this approach [29,44]. In terms of methods, what for many is a benefit—the flexibility it allows in terms of methods chosen [41]—can for others be quite daunting, especially when they are less experienced.

Since capacity building is such an important outcome envisaged for the program described, we turn briefly to this literature. It is a more extensive literature than on purveyor programs, but it can be argued that it contains much that can be useful to the latter. Kopf and Thayer [45], for example, reviewed the most successful capacity building initiatives of providers in the USA. They discovered that various external factors, apart from the content and quality of the capacity building program itself, influence significantly the effectiveness of these efforts. In particular:

1. Each organization has specific needs that should be taken into account by capacity building initiatives. Providers who work with organizations’ specific needs, instead of relying on formulas, get better results.

2. The better the understanding of an organization’s situation, history, and culture, the more effective the capacity building becomes.

3. Listening, communicating, and understanding an organization’s context is essential for effective capacity building.

4. Trust between the capacity-building initiative and the organization is essential for capacity building to occur. Both parties should feel free to communicate openly, to ask for help beyond the usual, and to listen and learn.

5. Capacity-building initiatives should spend sufficient time with organizations to obtain a good understanding of what the organization needs and how their skills and knowledge can be moulded to yield the most benefits for that organization.

This line of reasoning suggests that the nature of the relationship between purveyors, provider organizations, and implementers will have a strong influence on the success of these efforts. Furthermore, Kopf and Thayer’s findings [45] indicate that purveyors might benefit by becoming more flexible in the way they provide services to provider organizations. It would seem that a ‘one-size fits all’ approach might not be the most effective way to equip provider organizations to deliver interventions. Ideally, purveyors should spend sufficient time with each provider organization that they support to determine how their services could best be adapted to equip each specific organization. One of the external factors affecting provider organizations is the base capacity when adopting an intervention. It stands to reason that provider organizations with more capacity at the outset can benefit more from support than those who struggle with capacity issues. This is also the best indication of how supportive services should be adapted to deliver the best results. It will of course not be easy for purveyors to incorporate these observations into their daily activities, but we believe it to be worthwhile in building a sound evidence-based practice.

## Conclusion

It is clear from the literature that purveyors and intermediary organizations have an important role to play in the implementation of programs, especially in resource-poor settings. As we indicated above, they are a response to a perceived lack of capacity in provider organizations to deliver services with strength and fidelity, and as such they have an important role to fulfill. In the program that we studied, and many similar ones, this is what they set out to achieve. What would this ‘capacity’ mean in different human service settings, such as mental health, juvenile justice, public health, etc.? Would we recognize it when we see it? As basic human resource and organizational issues, our view is that the question can be answered affirmatively: after a purveyor has worked with a provider organization, the latter’s staff should be more skilled to deliver a specific intervention and have greater expertise than before, and the organization itself should have adequate governance and management structures to support its personnel. Whether it is possible to extract a more general program theory for purveyors across human services from this study is much more open to doubt, we believe. One, at least, would have to compare the effectiveness of the impact theories to which different purveyors subscribe, to be able to answer this question in some empirical way. A program-theory driven approach to evaluation nevertheless holds much promise in this regard.

## Endnote

^a^ Examples of intermediary and purveyor organizations that operate within this burgeoning field include: The Centre for Effective Services, based in Dublin, Ireland (http://www.effectiveservices.org/); Practice and Research Together, of Toronto, Canada (http://www.partontario.org/); The Evidence-based Prevention and Intervention Support Center (EPISCenter) of Pennsylvania State University (USA) (http://www.episcenter.psu.edu/); and the Connecticut Center for Effective Practice, based in New England, USA (http://www.chdi.org/ccep-initiatives.php). The in-text origin of this endnote is in the third paragraph under the Background section.

## Competing interests

The authors declare that they have no competing interests.

## Authors’ contributions

CO and JL conceived the study together. CO conducted the fieldwork, and JL provided input on the interpretation of the results. JL prepared the first draft of this manuscript, and both authors provided initial and final refinements. Both CO and JL read and approved the final manuscript.
